# Une cause rare d’abdomen aigu: l’appendagite épiploique

**DOI:** 10.11604/pamj.2018.30.267.5563

**Published:** 2018-08-08

**Authors:** Yassine Nhamoucha, Youssef Bouabdellah

**Affiliations:** 1 Service de Chirurgie Pédiatrique, CHU Hassan II, Fès, Maroc

**Keywords:** Abdomen aigu, enfant, appendagite épiploique, torsion, traitement, Acute abdomen, child, epiploic appendagitis, torsion, treatment

## Abstract

Primary epiploic appendagitis brings together torsions and primary inflammations of epiploic appendices. These pathological events are rare and, for a long period of time, have been exceptionally diagnosed in preoperative period. The progress of medical imaging allow, now, to avoid unnecessary surgical interventions. We report the case of a 14-year old child who was admitted to hospital with left iliac fossa pain evolving over six days and gradually worsening. He didn’t suffer from transit disorders or associated urinary functional symptoms and he was afebrile. Abdominal palpation showed right iliac fossa tenderness. He was overweight with a BMI of 29. Laboratory tests didn’t show any inflammatory syndrome. Cytobacteriologic examination of the urine was negative. Abdominal x-ray without treatment didn’t show any hydroaeric level or calcification in urinary tract projections. CT scan objectified fat infiltration in the coeco-appendicular junction, with shuttle image containing fat surrounded by hyperdense ring, suggesting a torsion of the epiploic appendice. The child underwent symptomatic treatment with good clinical evolution.

## Image en médecine

L’appendagite épiploïque primitive regroupe les torsions et les inflammations primitives des appendices épiploïques. Ces événements pathologiques considérés comme rares, ont longtemps été exceptionnellement diagnostiquées en période préopératoire. Les progrès de l’imagerie médicale permettent, désormais, d’éviter des interventions chirurgicales inutiles. C’est ce qu’illustre l’observation présentée ici. Il s’agit d’un enfant de 14 ans, sans antécédents pathologiques notables, qui s'était présenté aux urgences pédiatriques de l’hôpital, pour des douleurs de la fosse iliaque gauche évoluant depuis six jours et d’aggravation progressive. Il ne souffrait ni de troubles du transit, ni de signes fonctionnels urinaires associés et était apyrétique. La palpation abdominale mettait en évidence une défense de fosse iliaque droite. On notait, par ailleurs, un surpoids avec un IMC à 29. Biologiquement, la CRP était à 11 mg/l (N < 1), les leucocytes à 10.000 par millimètre cube à prédominance polynucléaires neutrophiles. L’examen cytobactériologique des urines était négatif. La radiographie de l’abdomen sans préparation ne mettait en évidence ni niveau hydroaérique, ni calcification en projection des voies urinaires. L’échographie abdominale a montré au niveau du flanc droit une petite masse hyperéchogène, ovoïde, non dépressible, entourée d’un halo hypoéchogène contenant en son sein quelques vaisseaux fins sans dilatation des voies urinaires ni d’épanchement intra-abdominal. Le complément scannographique révélait une infiltration de la graisse à la jonction coeco-appendiculaire, avec image graisseuse en navette entourée d’un anneau hyperdense, évocatrice d’une torsion d’appendice épiploïque. Il était décidé de traiter le malade par des antalgiques, des antispasmodiques, un mois plus tard le patient n’avait plus de douleur abdominale spontanée ou provoquée.

**Figure 1:**
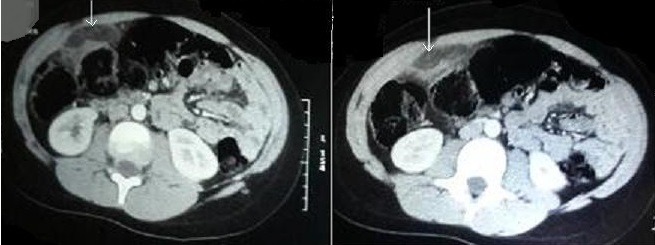
tomodensitométrie abdominale avec injection du produit de contraste: la flèche montre la nécrose d’une frange épiploïque (appendagite) en avant de la paroi du côlon gauche

